# Supplementary material for: FRAME—Monte Carlo model for evaluation of the stable isotope mixing and fractionation
Source: PLoS One. 2022 Nov 28;17(11):e0277204. doi: 10.1371/journal.pone.0277204 (PMC9704640; doi:10.1371/journal.pone.0277204)
Supplement: S1 Appendix — (PDF) [file pone.0277204.s001.pdf]

Supporting Information to: FRAME - Monte Carlo model for evaluation of the stable isotope mixing and fractionation

Maciej P. Lewicki[1*], Dominika Lewicka-Szczebak[2], Grzegorz Skrzypek[3],

1 Institute of Nuclear Physics, Polish Academy of Sciences, Krakow, Poland
2 Institute of Geological Sciences, University of Wrocław, Wrocław, Poland
3 West Australian Biogeochemistry Centre, School of Biological Sciences, The University of Western Australia, Perth, Australia

* maciej.lewicki@ifj.edu.pl

A Appendix

A.1 Algorithm details

FRAME introduces a new mathematical treatment of isotope mixing problems, which is unique in two main aspects:

- **Studied system may undergo a process of fractionation**, meaning that the isotope composition of the mixture is a result of not only the mixing of predefined sources (each characterised by its isotope composition signature), but also some chemical or physical time-dependent process (or processes). Thus, the linear model of pure mixing is modified by an additional term. This topic is explored in detail in Sec. **??** and **??**.
- **Input data are treated as a range** of equally probable values instead of a single value with uncertainty. The introduction of such a feature has a number of practical consequences (discussed in Sec. **??**), however, it also results with substantial changes in the mathematical formulation of the model. In particular, the likelihood function needs to be modified to account for the non-normal probability distribution of values characterising mixing components. The following section describes the details and derivation of the new, *modified likelihood function.*

A.1.1 Modified likelihood function

The standard form of the likelihood function is given as a Gaussian:

$$L(x) = \frac{1}{\sqrt{2\pi}\sigma} \exp\left(\frac{-(x-\mu)^2}{2\sigma^2}\right) \tag{1}$$

with the width σ standing for combined uncertainty of model parameters (quadratic sum of uncertainties of isotopic signatures and auxiliary parameters), x stands for the measured isotopic signature of the sample and μ is the model output value, which most general definition can be written as:

$$\mu = \sum_{i=1}^{m} f_i \, \boldsymbol{S}_i + \mu_{\text{aux}}(\boldsymbol{A}, \boldsymbol{r}) \tag{2}$$

The variable μ can be rewritten as

$$\mu = \mu' + \alpha S_0 \tag{3}$$

where $\alpha = \frac{\partial \mu}{\partial S_0}$ and μ' does not depend on S_0 (linear dependence of μ on S_0 was assumed). Let us consider the isotopic signature of the source S_0 as a range of equally probable values, thus being represented as a flat probability distribution in the range of: $(S_0 - \Delta S_0, S_0 + \Delta S_0)$. The likelihood function with such a modification will be derived and then the equation will be generalized to account for a flat distribution of all S_i sources. For convenience, the apostrophe in μ' will be dropped and written simply as μ.

Since the source isotopic signature can be any value in the range $(S_0 - \Delta S_0, S_0 + \Delta S_0)$ the basic likelihood function (Eq. 1) will become an integral:

$$\int_{S_0-\Delta S_0}^{S_0+\Delta S_0} \frac{1}{\sqrt{2\pi}\sigma} \exp\left(\frac{-(x+\alpha S-\mu)^2}{2\sigma^2}\right) \mathrm{d}S =$$

$$= \frac{1}{\sqrt{2\pi}\sigma} \exp\left(\frac{-(x-\mu)^2)}{2\sigma^2}\right) \int_{S_0-\Delta S_0}^{S_0+\Delta S_0} \exp\left(\frac{-(\alpha^2 S^2 + 2\alpha S x - 2\alpha S\mu)}{2\sigma^2}\right) \mathrm{d}S =$$

$$= \frac{1}{\sqrt{2\pi}\sigma} \exp\left(\frac{-(x-\mu)^2}{2\sigma^2}\right) \sqrt{\frac{\pi}{2}}\, \frac{\sigma}{\alpha} \exp\left(\frac{(\mu-x)^2}{\sqrt{2\sigma^2}}\right) \left[\mathrm{erf}\left(\frac{\alpha S-\mu+x}{\sqrt{2\sigma^2}}\right)\right]\Bigg|_{S_0-\Delta S_0}^{S_0+\Delta S_0} =$$

$$= \frac{1}{2\alpha}\left(\mathrm{erf}\left(\frac{\alpha(S_0+\Delta S_0)-\mu+x}{\sqrt{2\sigma^2}}\right) - \mathrm{erf}\left(\frac{\alpha(S_0-\Delta S_0)-\mu+x}{\sqrt{2\sigma^2}}\right)\right)$$

and the normalization can be calculated with:

$$\int_{-\infty}^{\infty} \frac{1}{2\alpha}\left(\mathrm{erf}\left(\frac{\alpha(S_0+\Delta S_0)-\mu+x}{\sqrt{2\sigma^2}}\right) - \mathrm{erf}\left(\frac{\alpha(S_0-\Delta S_0)-\mu+x}{\sqrt{2\sigma^2}}\right)\right) =$$

$$= \frac{1}{\alpha}\left(\alpha(S_0-\Delta S_0) - \alpha(S_0+\Delta S_0)\right) = -2\Delta S_0$$

Finally we obtain:

$$L(x+\alpha S) = \frac{1}{4\alpha\Delta S_0}\left(\mathrm{erf}\left(\frac{\alpha(S_0-\Delta S_0)-\mu+x}{\sqrt{2\sigma^2}}\right) - \mathrm{erf}\left(\frac{\alpha(S_0+\Delta S_0)-\mu+x}{\sqrt{2\sigma^2}}\right)\right)$$

Such a function can be easily generalized to the finite flat distribution of all contributing sources:

$$L(x) = \frac{1}{4\alpha\Delta S_0}\left[\mathrm{erf}\left(\frac{\sum_i(\alpha_i(S_0-\Delta S_0))-\mu+x}{\sqrt{2\sigma^2}}\right) - \mathrm{erf}\left(\frac{\sum_i(\alpha_i(S_0+\Delta S_0))-\mu+x}{\sqrt{2\sigma^2}}\right)\right]$$

which can be expressed by invoking the original definition of x and α_i (multiplicative constants were dropped, as absolute normalization is irrelevant):

$$L(\boldsymbol{\mu}|\boldsymbol{\mu},\boldsymbol{\sigma}) \propto \prod_{i\,\in\,\text{isotopes}} \frac{1}{\Delta_i}\left[\mathrm{erf}\left(\frac{-\Delta_i+\mu_i-x_i}{\sqrt{2\sigma_i^2}}\right) - \mathrm{erf}\left(\frac{\Delta_i+\mu_i-x_i}{\sqrt{2\sigma_i^2}}\right)\right]$$

$$\text{where} \qquad \Delta_i = \sum_{j\in\text{sources}} \frac{\partial\mu_i}{\partial S_{ij}}\Delta S_{ij}$$

Δ_i is dependent on fractions f_i (and possibly other auxiliary variables), thus it must be evaluated in each MC iteration.

$$f(x) = \frac{1}{\sqrt{2\pi(\sigma^2+\Delta^2)}} \exp\left(\frac{-x^2}{2(\sigma^2+\Delta^2)}\right), \quad g(x) = \frac{1}{4\Delta}\left(\mathrm{erf}\left(\frac{x+\Delta}{\sqrt{2}\sigma}\right) - \mathrm{erf}\left(\frac{x-\Delta}{\sqrt{2}\sigma}\right)\right)$$

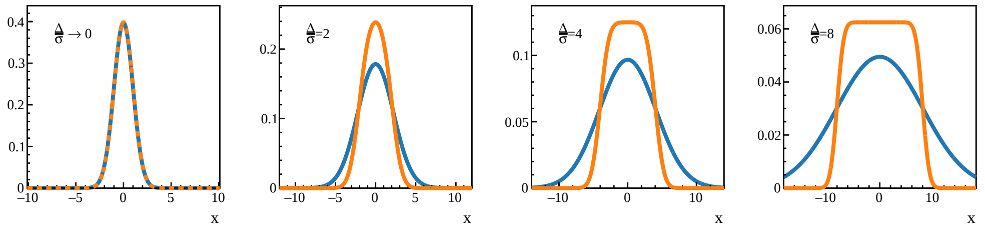

Figure S1. Comparison of gaussian-like and erf-like ($f(x)$ and $g(x)$ respectively) function shapes depending on the relation between Δ and σ. Note that in the limit of vanishing Δ functions g and f become close to equal, however function g is indefinite at $\Delta = 0$, so FRAME switches back to gaussian-like likelihood function in these cases.

B User guide

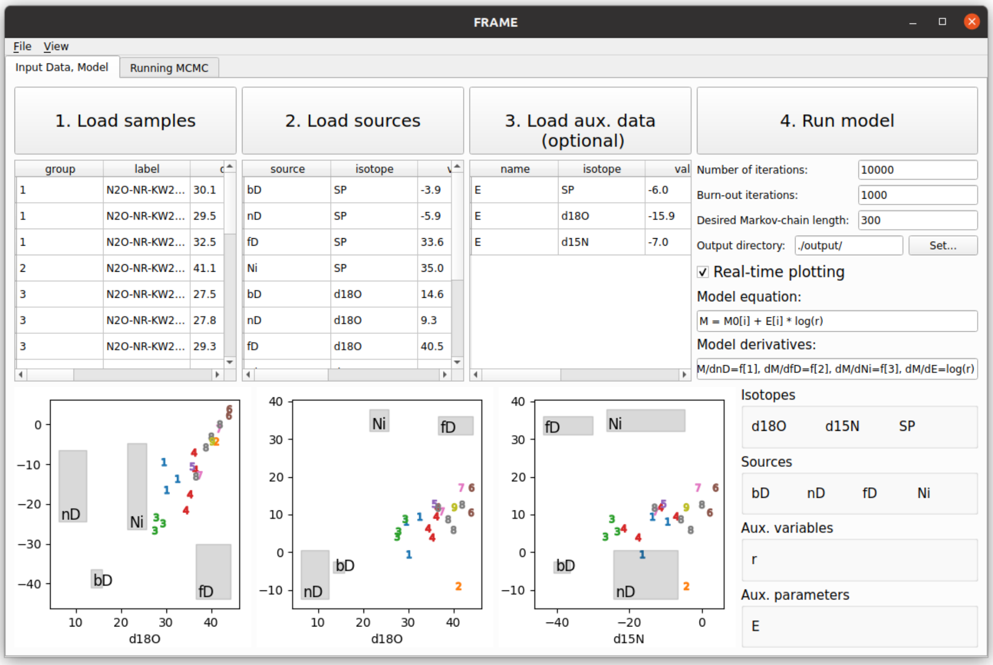

Figure S2. The main interface of the program.

Figure S2 presents the main interface of the program, in which the input data is loaded and simualtion parameters are set. All the input files for the model should be prepared in .csv format. All the example input files used for simple examples (Section 3) and for the case studies (Section 5) described in the paper are accessible on the FRAME website (https://malewick.github.io/frame/).

1. Load samples
   First, the .csv file with samples data should be uploaded. The stable isotope signatures of the samples should be introduced into the model by defining the following columns:
   - **group**- the following number for samples group representing common samples class

or series of measurements. In other words, the group is a pool of repetition of the same sample and only one solution/result will be calculated for the group without calculation of individual fractions for each sample

- **label** - identification of individual samples which is displayed on the graphs generated by the model.
- **stable isotopic signatures** in two columns for 2D model and three columns for 3D model - with the user-defined name as the heading (e. g., in plain text "d18O" or using unicode characters "$\delta^{18}O$") and δ-values in per-mill (‰) as entries for each sample.
- **standard deviation of stable isotopic signatures** in two columns for 2D model and three columns for 3D model - with heading as `stdev(isotopic signature)`, e.g. `stdev(`$\delta^{18}O$`)`, and the values of measurement uncertainties as entries for each sample.
  See examples: `NO3data.csv, H2Odata.csv, NITRITEdata.csv, NO2data.csv`

2. Load sources
Second, the .csv file with sources data should be uploaded. The isotopic characteristic of the sources should be introduced into the model by defining the following columns:

- 'source' – name or abbreviation of the source
- isotopic signatures in two columns for 2D model and three columns for3D model - with the name of isotopic signature as the heading, identical to the headings defined in samples file (e.g.$\delta^{18}O$) and mean isotopic δ values as entries for each source. In case of defining the range of equally probable values representing the source the mean value = (range max + range min)/2 should be given.
- spread of the isotopic signature - e.g. spread($\delta^{18}O$) – the spread of the isotopic range of the particular source, i.e., if spread = (range max - range min)/2, the range is defined as mean value ± spread. All the values within this range have equal probability. In case of one well-determined mean value of the highest probability , the spread should be entered as zero.
- standard deviation of the isotopic signature – e.g. stdev($\delta^{18}O$) - standard deviation representing the analytical uncertainty of the isotopic analysis of the source values – this uncertainty is added to the mean value ± spread range as a margin with Gaussian likelihood (see Figure S1). By inserting 0 as standard deviation you can omit this uncertainty.
  See examples: NO3_sources.csv, H2O_sources.csv, nitrite_sources.csv, NO2_sources.csv
- Optional: load auxiliary data
  Third, the .csv file with fractionation data can be uploaded, this is optional, if omitted the model applies only mixing calculations. With this file the additional parameter associated with isotopic fractionation can be added, by defining the following rows:
  - row 1 - define the model equation including auxiliary data, where: M0[i] represents the final isotopic mixture of all sources before fractionation; capital letters [i] represent the parameters, e.g. the isotopic fractionation factors; letter "r" represents the unknown quantity, e.g. the residual fraction (use r[0], r[1], ... in case of more unknowns). See practical examples of equations below and in example files.
  - row 2 - define the columns for entering data of the defined parameters: 'name' - indicate which parameter (capital letter); indicate the following isotopic values which are used by the model with identical headings as used in samples and sources file, (e.g. $\delta^{18}O$); spread (e.g. spread($\delta^{18}O$)) - analogically as in source file defines the spread of the range; stdev (e.g. spread($\delta^{18}O$)) - analogically as in source file defines the analytical uncertainty.
    - the following rows - for the entries of values for the above defined values

Examples of model entries for the fractionation equations (first line of the auxiliary data file):

- Open system fractionation
  M0[i] + E[i]*r
  where M0[i] stands for the isotopic signature of initial mixture before fractionation: M0[i]=f[0]*S[i][0]+f[1]*S[i][1]+f[2]*S[i][2]... - where f is the fraction of each source and S[i] is the characteristic isotopic signature of each source (note that first source is denoted with 0 not 1), E[i] stands for isotopic fractionation factor and r for the residual unreacted fraction.
  In the case when only one of the contributing sources undergoes fractionation it should be indicated with the respective fraction, e.g.: M0[i] + f[0]*E[i]*r
  In case for different isotopic fractionation for different sources, you may further specify this as, e.g.: M0[i] + f[0]*E[i]*r+f[1]*A[i]*r
- Closed system (Rayleigh-type) fractionation
  M0[i] + E[i]*log(r)
  M0 stands for the isotopic signature of initial mixture before fractionation: M[0]=f[0]*S[i][0]+f[1]*S[i][1]+f[2]*S[i][2]... - where f is the fraction of each source (note that first fraction is denoted with 0 not 1) and i is the characteristic isotopic signature of each source, E[i] stands for isotopic fractionation factor and r for the residual unreacted fraction. Note that the natural logarithm is denoted with log in the model language.
  In the case when only one of the contributing sources undergoes fractionation it should be indicated with the respective fraction, e.g.: M0[i] + f[0]*E[i]*log(r)
- Equilibrium fractionation
  M0[i]*(1-r*A[i]) + D[i]* r * A[i]
  where M stands for the final isotopic signature, M0 stands for the isotopic signature of initial mixture before fractionation: M[0]=f[0]*S[i][0]+f[1]*S[i][1]+f[2]*S[i][2]... - where f is the fraction of each source and i is the characteristic isotopic signature of each source, D[i] stands for isotopic signature after equilibration and r for the equilibrated fraction. The additional parameter A[i] can be defined for the isotope undergoing equilibration as 1 and for the isotope which is not undergoing equilibration as 0, or any other values depending on the equilibration ratio between various isotopes.

See examples: NO3frac.csv, H2Ofrac.csv, NITRITEfrac.csv, NO2frac.csv

You can also save your current configuration into xml file, which can later be conveniently loaded to resume work (`File→Save, File→Load`).

Once you run the model you will see the output console informing you about the state of the calculation and three additional plotting canvases will appear (see Fig. S3).

- Figure 1. Time-series of entries accepted by the Metropolis-Hastings algortihm, that build the Markov chain. The burnout period is marked with a dashed lined.
- Figure 2. On the diagonal there are histograms showing distributions of evaluated variables. Panels above the diagonal show the correlation between the variables and panels below the diagonal show the same correlation, but evaluated as a single number.
- Figure 3. For each accepted set of variables the isotope mixture is evaluated and plotted as a path.

These plots serve the purpose of real-time quality assessment during the calculation and it is advised to always begin analyses by running a couple of test samples to see if everything works as expected. However, when running the analysis for multiple samples, it is advised to turn off the on-line plotting to reduce the computation time.

Also, when the model is run the FRAME interface switches to a new tab, which contains the output console and the progress bar to inform you about the status of the computation.

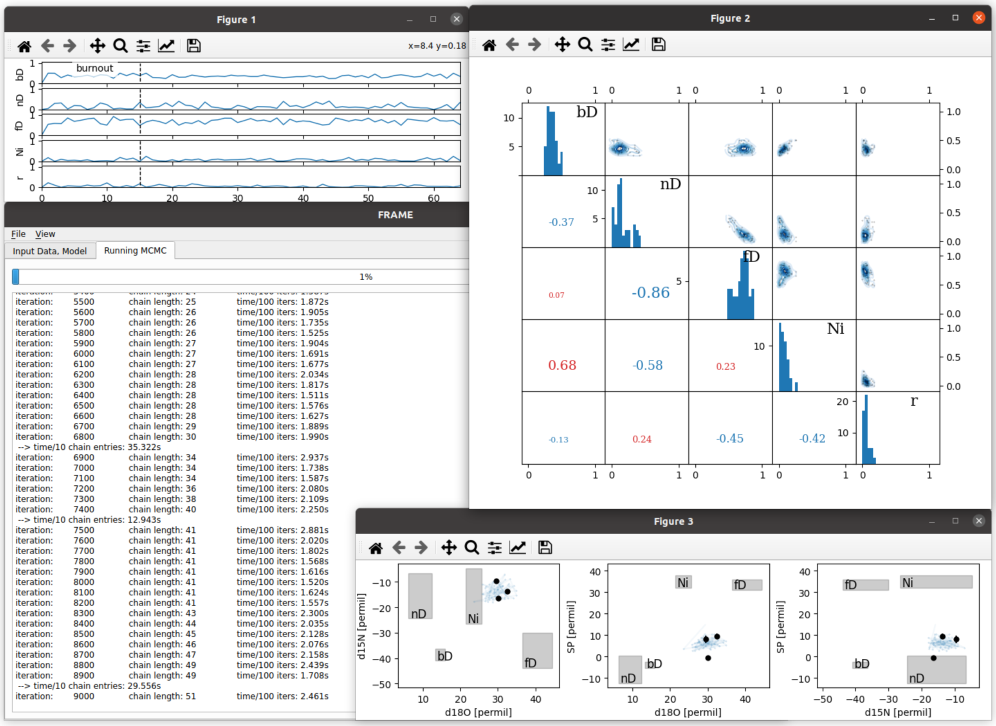

Figure S3. The view of the running simulation.

When the run is finished the results are saved in the given directory in the folder (named with the combined input file names) as results.csv that contains the calculated final values for each unknown parameter (in the following columns) for each sample group (in the following rows). The following values are determined: - mean - median - standard deviation (given as 1 σ) - lim low - lower limit of 68% confidence level (calculated as shown in Sect. 2.3) - lim up - upper limit of 68% confidence level (calculated as shown in Sect. 2.3) The 3 figures listed above are also saved in the output folder.

The interface can be practically tested using the files prepared for the example datasets presented in the Section **??** which are available at https://github.com/malewick/frame:

-2D model for determination of nitrate sources and fractionation (**??**): sources (NO3_sources.csv), measured values of the mixture (NO3_data.csv), fractionation (NO3_frac.csv)

-2D evaporation model for waters (??): sources (H2O_sources.csv), measured values of the mixture (H2O_data.csv), fractionation (H2O_frac.csv)

- 2D model for determination of nitrite pathways (??: sources (nitrite_sources.csv), measured values of the mixture (nitrite_data.csv), fractionation (nitrite_frac.csv)

- 3D model for N2O source partitioning and quantification 568 of N2O reduction (??): sources (N2O_sources.csv), measured values of the mixture (N2O_data.csv), fractionation (N2O_frac.csv)

C More examples with FRAME

C.1 Many indistinguishable sources (2D)

Here we show the example model outputs in case of too many indistinguishable sources are introduced into the model and it fails in finding the solution (Figs. (S4, S5, S6, S7).

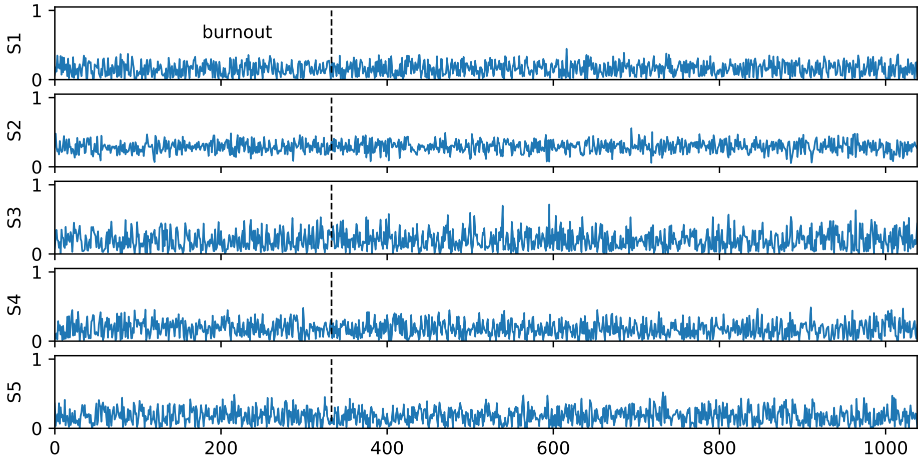

Figure S4. Mixing configurations (f_1, f_2, f_3) from all model iterations, which fulfilled the Metropolis condition – the Markov chain. A number of initial iterations is discarded in order for the model to stabilize (burnout, left to the dashed line), reaching the region of maximum likelihood. In MCMC simulations it is crucial that the stabilization is indeed reached, therefore such plot is a key tool for quality assessment. In case the stabilization is not achieved, the plot would display large variations, exceeding the iteration-by-iteration fluctuations, as well as longer trends would appear, persisting for a > 1 number of iterations.

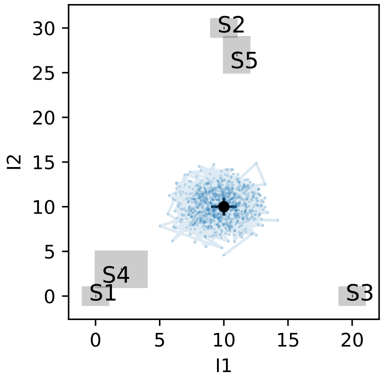

Figure S5. The path of consecutive entries stored in the Markov chain plotted in I_1, I_2 plane. Each dot represents a model value $\boldsymbol{\mu}$ calculated for each of mixing configurations $(f_1, f_2, f_3, f_4, f_5)$ and the line connects consecutive entries. The measurement is denoted with a black dot and error bars, while the sources and their spread are represented with shaded rectangles.

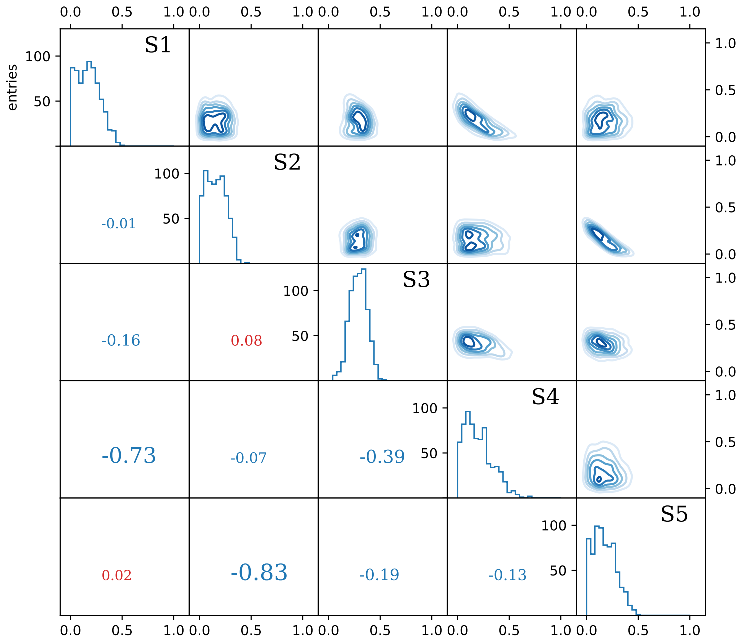

Figure S6. Histograms (on the diagonal) calculated from the variables building the Markov chains along with their correlations as contour-plots (top-right) and correlation coefficients (bottom-left).

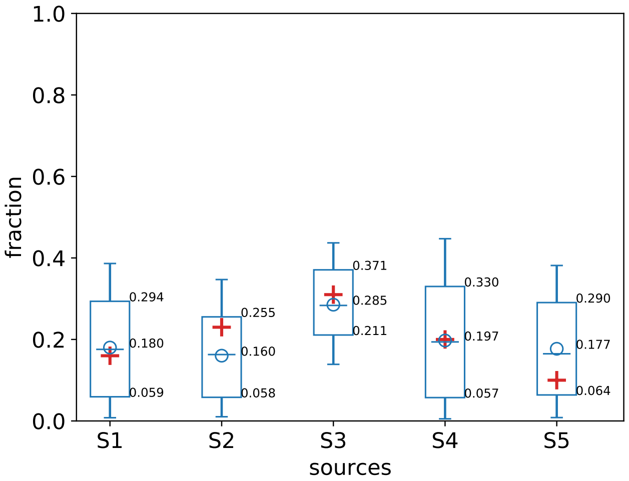

Figure S7. The candle plots illustrating the results of the simulation. The blue circle represents the mean, the horizontal line stands for median, the box shows the boundaries of the middle quartiles, and the whiskers enclose 95% of the distribution.

C.2 Measurement on the edge (2D)

Here we show the example model outputs in case of measured values of the mixture are situated on the edge of possible mixing area defined by the sources (Figs. (S8, S9, S10, S11).

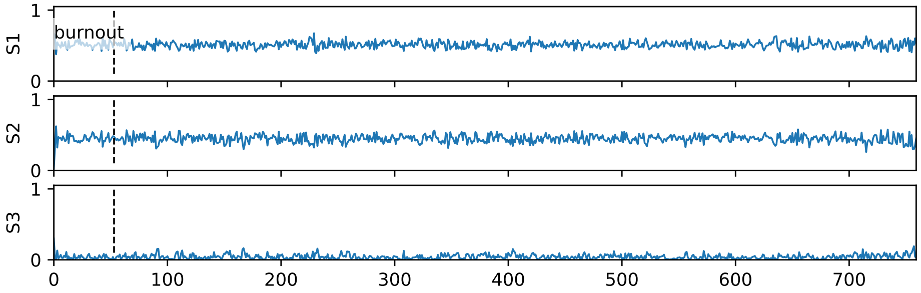

Figure S8. Mixing configurations (f_1, f_2, f_3) from all model iterations, which fulfilled the Metropolis condition – the Markov chain. A number of initial iterations is discarded in order for the model to stabilize (burnout, left to the dashed line), reaching the region of maximum likelihood. In MCMC simulations it is crucial that the stabilization is indeed reached, therefore such plot is a key tool for quality assessment. In case the stabilization is not achieved, the plot would display large variations, exceeding the iteration-by-iteration fluctuations, as well as longer trends would appear, persisting for a > 1 number of iterations.

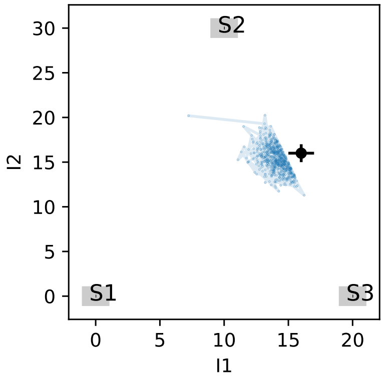

Figure S9. The path of consecutive entries stored in the Markov chain plotted in I_1, I_2 plane. Each dot represents a model value $\boldsymbol{\mu}$ calculated for each of mixing configurations (f_1, f_2, f_3) and the line connects consecutive entries. The measurement is denoted with a black dot and error bars, while the sources and their spread are represented with shaded rectangles.

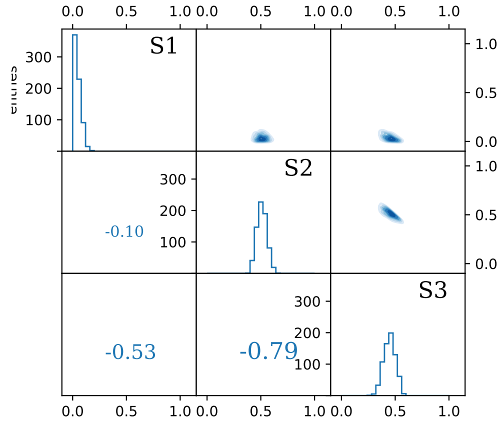

Figure S10. Histograms (on the diagonal) calculated from the variables building the Markov chains along with their correlations as contour-plots (top-right) and correlation coefficients (bottom-left).

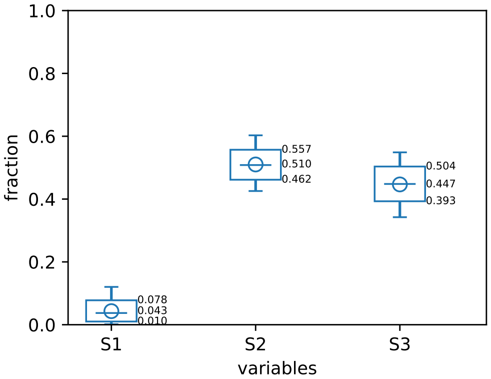

Figure S11. The candle plots illustrating the results of the simulation, comparing with the true values (drawn in red). The blue circle represents the mean, the horizontal line stands for median, the box shows the boundaries of the middle quartiles, and the whiskers enclose 95% of the distribution.
