# Supplementary material for: FRAME—Monte Carlo model for evaluation of the stable isotope mixing and fractionation
Source: PLoS One. 2022 Nov 28;17(11):e0277204. doi: 10.1371/journal.pone.0277204 (PMC9704640; doi:10.1371/journal.pone.0277204)
Supplement: Attachment — Submitted filename: review response.pdf [file pone.0277204.s002.pdf]

Dr Matheus C. Carvalho

Academic Editor

PLOS ONE

21 September 2022

Dear Dr Carvalho,

We are pleased to resubmit our manuscript (PONE-D-21-35676) entitled " FRAME - Monte Carlo model for evaluation of the stable isotope mixing and fractionation" after minor revision for consideration in PlosONE journal.

Please see our detailed responses in green below the reviewers' comments and the changes marked in the PDF file. All comments are rather editorial, or these are requests for additional information or clarifications. The review helped to improve the manuscript but did not change our conclusions or the proposed calculation model.

We look forward to hearing your comments on this version of the paper.

Sincerely yours,

Maciej Lewicki

Corresponding author on behalf of all authors

Review response

Please see in green our answers to all raised points.

Editor comments:

When submitting your revision, we need you to address these additional requirements.

1. Please ensure that your manuscript meets PLOS ONE's style requirements, including those for file naming. The PLOS ONE style templates can be found at https://journals.plos.org/plosone/s/file?id=wjVg/PLOSOne_formatting_sample_main_body.pdf and https://journals.plos.org/plosone/s/file?id=ba62/PLOSOne_formatting_sample_title_authors_affiliations.pdf

We have reviewed the style guidelines, as well as the file names and all seems to be in order, following journal requirements. Please let us know if we are missing some specific formatting.

2. Please upload a new copy of Figure 14 as the detail is not clear. Please follow the link for more information: https://blogs.plos.org/plos/2019/06/looking-good-tips-for-creating-your-plos-figures-graphics/" https://blogs.plos.org/plos/2019/06/looking-good-tips-for-creating-your-plos-figures-graphics/

Indeed the resolution of Figure 14 in the PDF file produced during the manuscript submission is very poor, similarly the quality of a few other figures is unsatisfactory. However, the resolution and readability of the original tiff files submitted by us with the manuscript, are very good . Probably, compression of the figure during PDF production was reducing the figure resolution too far. Thus, it is not clear if anything can be done on our side to improve the figure quality. Please let us know if there is anything that we could try to improve the compressed figure quality.

3. Please ensure that you refer to Figures 9, 14, 15, 16, 17, 18, 19, 20, 21, 22, and 23 in your text as, if accepted, production will need this reference to link the reader to the figure.

This has been corrected; each figure is now referred to in the text.

4. We note that Figures 5 and 14 in your submission contain copyrighted images. All PLOS content is published under the Creative Commons Attribution License (CC BY 4.0), which means that the manuscript, images, and Supporting Information files will be freely available online, and any third party is permitted to access, download, copy, distribute, and use these materials in any way, even commercially, with proper attribution. For more information, see our copyright guidelines: http://journals.plos.org/plosone/s/licenses-and-copyright.

Figures 5 and 14 have been generated using our software, which has been created by the authors of this manuscript. This software is an original contribution and it is not subject to a copy rights of third parties.

5. We note you have included a table to which you do not refer in the text of your manuscript. Please ensure that you refer to Tables 1, 2, 3, 4, 6, 7, 8 and 9 in your text; if accepted, production will need this reference to link the reader to the Table.

This has been corrected; each figure is now referred to in the text.

6. Please review your reference list to ensure that it is complete and correct. If you have cited papers that have been retracted, please include the rationale for doing so in the manuscript text, or remove these references and replace them with relevant current references. Any changes to the reference list should be mentioned in the rebuttal letter that accompanies your revised manuscript. If you need to cite a retracted article, indicate the article's retracted status in the References list and also include a citation and full reference for the retraction notice.

We have revised our reference list; it is complete and correct.

<u>Reviewers' comments:</u>

<u>Reviewer #1:</u> General comments
This paper describes a computational model for unraveling mixing and isotopic fractionation that occurs various environmental/geochemical/ecological settings. Since stable isotope ratios often show distinct values between different source materials, isotope ratios of a sample and its potential sources are compared to estimate contribution from each source. However, it is not always the case that proportions of the sources are algebraically calculated because of too many potential sources or isotopic change (fractionation) during chemical/physical conversion of target compounds after mixing. In addition, error associated with the source partitioning is not easy to derive. The authors developed a convenient and flexible tool that can calculate mixing rate of sources with probability distributions and effect of other factors such as isotopic fractionation. They also present many examples showing how the tool can resolve the mixing problems of real conditions appropriately.
Although I cannot fully evaluate the mathematical theory part because it is outside my area of expertise, I think this paper will bring great benefits to researchers using stable isotopes to elucidate material cycles in a variety of disciplines. I recommend its publication after addressing following points.

Thank you very much for your time devoted to reviewing our manuscript and for your positive opinion and important suggestions. We have corrected the manuscript according to your comments, please find our response to the specific points below.

Specific comments
Abstract. The authors write "this tool can also be useful for non-stable isotope studies", but no example of such application is listed. It would be better to add some ideas.

Thank you for this comment. Our intention was to indicate that this presented tool may also be potentially used for solving other mixing problems besides stable isotope studies. However, since the authors specialise in this area, we have only suitable examples from our area of expertise, and so far are not able to add examples from non-stable isotope studies. Therefore, we admit that this statement should be removed from the abstract; we only indicate this potential application in the outlook and conclusions section.

L17–18. I think there was an attempt at estimation of errors in source partitioning with consideration of isotopic fractionation although the approach was simpler than the present study (Toyoda, S., Yano, M., Nishimura, S., Akiyama, H., Hayakawa, A., Koba, K., et al. (2011). Characterization and production and consumption processes of N2O emitted from temperate agricultural soils determined via isotopomer ratio analysis. Global Biogeochem. Cycles, 25, GB2008).

Thank you very much for this suggestion. We regret omitting this important paper in our literature review. Indeed, this study was the first in N2O studies, where estimation of errors was taken into account. This reference with the relevant information was added to the introduction (in the paragraph regarding N2O modelling) lines: 44-48.

L33–43. In the work of Lewicka-Szczebak et al. (2020) they compared two cases: 1) N2O reduction by denitrification first occurs and then mixed with N2O from other sources, 2) N2O reduction occurs after mixing. But in the present paper, it seems only the case 2) is considered. Can the developed tool also simulate the case 1)?

Thank you very much for this question; indeed, we missed to explain this in the manuscript.

Yes, the tool can simulate both cases, this can be done by entering the proper equation describing the fractionation process.

For reduction first (case 1)

$$M = f_{bD}(S_{bD} + A\ln(r)) + f_{nD}S_{nD} + f_{fD}S_{fD} + f_{Ni}S_{Ni}$$

For reduction after mixing (case 2)

$$M = f_{bD}S_{bD} + f_{nD}S_{nD} + f_{fD}S_{fD} + f_{Ni}S_{Ni} + A\ln(r)$$

Here, for simplicity, we described case 2 only for N2O. The same calculation algorithm was used in the previous paper, but now we build it into a software with a graphical interface improving also propagation of errors and redesigning method for accounting the range of values for the defined sources.

We agree that to be precise and provide full information it is important to inform readers that we can take into account the fractionation of the whole mixture as well the fractionation of only one source before mixing. This information has been added in Section 3.3, where various types of possible fractionation with respective equations are discussed.

L129. Correct typo (1,m]

This typo has been corrected.

L422–425. Although it is not critical in the context, it would be better to note that overall isotope fractionation is not always equal to simple sum of fractionation for the two step in

order to avoid misunderstanding.

This is correct. This description was very simplified and actually not needed here because, as the reviewer suggests, it is not particularly relevant in this context. We further revised this description and gave only a summary fractionation for the entire process to avoid misunderstanding.

Figure 1 caption, the last line, “The bottom right panel”. LEFT panel?
Thank you very much for your careful reading! This mistake has been corrected.

Also please add titles of axes, same for other figures.

In the standard FRAME output plots showing the distributions and correlations of variables estimated in the model, the horizontal axes represent fractions and vertical axes represent either fractions (in case of correlations) or counts (in case of histograms). After testing multiple solutions, we decided not to include axis descriptions on these plots, because they significantly reduce readability. Instead, a careful description of the axes was moved to the figure caption. Moreover, as these plots are automatically generated by the FRAME software, it would be very challenging to implement a “one-fit-all” solution for axis description.

Figure 2. Some characters in the figure do not correspond to those explained in the caption. I think the axis titles “X” and “Y” must be “I1” and “I2”, respectively. Also “M” means x?

The labels in the plot were misleading. The figure has been changed accordingly.

Figure 3. I cannot understand why one can tell “the stabilization is achieved” from the figure. To my eyes, oscillating feature is almost the same between before and after the dashed line.

A following explanation was added to the figure caption:
In this example, the simulation begins with fractions already close to the equilibrium value, thus the stabilization is achieved virtually instantly and no visible change in oscillations is observed in dependence on the iteration number.

Figures 16–19. It is confusing that caption says the authors consider only three endmembers while five sources are plotted in the figure.

This mistake has been corrected. Thank you for remarking on this.

<u>Reviewer #2:</u> The paper presents a working open source framework for stable isotope analisys.
The software is user friendly and shows some original features in respect to other similar works. In particular it can consider fractination process besides isotope mixing.

The paper is quite well written and clear. There are just some minor notes and suggestions for authors:

Thank you very much for your time devoted to reviewing our manuscript and for your positive feedback and helpful suggestions. Please find our responses to your detailed comments below.

- The examples are usually well presented and references are always given. However, there can be some difficulties for readers to get a rapid introduction to them. A small introductiion paragraph for each example, what is the addressed problem and what can be gained with isotope analisys, can increase readability.

We have rewritten the example case studies paragraphs, expanding a bit the general introduction to each case to improve clarity.

- The software works flawlessly on Linux/wine, however it is not well clear what is the right combination of data/source/model for each example. Here also an introduction would be welcome. A comment part in the data file would be very useful.

We have added a description of each case in the supplement (appendix B) with an indication of which files should be applied for which case.

- In the last paragraph of the comment of Figure 1, I think there is an error: right --> left.

Yes, thank you for your careful reading! This mistake has been corrected.
