# Supplementary material for: FRAME—Monte Carlo model for evaluation of the stable isotope mixing and fractionation
Source: PLoS One. 2022 Nov 28;17(11):e0277204. doi: 10.1371/journal.pone.0277204 (PMC9704640; doi:10.1371/journal.pone.0277204)
Supplement: Attachment — Submitted filename: revision1_DLS.docx [file pone.0277204.s003.docx]

**Dr Viacheslav Kovtun**

**Academic Editor**

**PLOS ONE**

18 October 2022

Dear Dr Kovtun,

We are pleased to resubmit after the second review process our manuscript (PONE-D-21-35676 ) entitled “ FRAME - Monte Carlo model for evaluation of the stable isotope mixing and fractionation” after additional few minor corrections for consideration in PlosONE journal.

Please see our responses in green below the reviewers’ comments and the changes marked in the PDF file. The two corrections pointed out by the reviewer 1 are purely editorial and have been included in the manuscript.

We look forward to the acceptance of our manuscript.

Sincerely yours,

Maciej Lewicki

Corresponding author on behalf of all authors

**Review response**

*Please see in green our answers to the raised points.*

**Reviewer #1:**

In the revised manuscript, the problems I pointed out have been improved. I think it is now acceptable after a few minor corrections below.

*Thank you very much for checking our manuscript again. We have corrected the mistakes you pointed out as requested.*

L12–13. I cannot understand why the authors revised this phrasing while they did not change the words at L4. I cannot make sense out of the phrase "n elements stable isotope composition determination"

*This is right, this phrasing was too complex, we changed this to use consequently ‘n isotopes analysed in a sample’*

L50. Typo. “signature”

*It was corrected.*
